# Functional outcomes of surgical treatment of varicocele in infertile men: Comparison of three techniques

**DOI:** 10.1016/j.amsu.2022.103937

**Published:** 2022-06-04

**Authors:** Yassine Ouanes, Moez Rahoui, Kays Chaker, Mahdi Marrak, Mokhtar Bibi, Kheireddine Mrad Dali, Ahmed Sellami, Sami Ben Rhouma, Yassine Nouira

**Affiliations:** Urology Department, La Rabta Hospital, Tunis, Tunisia

**Keywords:** Infertility, Varicocele, Semen analysis, Surgery

## Abstract

**Introduction:**

Among identified causes of male infertility, varicocele holds an important place and is significantly associated with sperm quality deterioration. Surgical management of this condition leads to an improvement in the sperm count and an increase in the spontaneous pregnancy rate.

**Objective:**

The goal of this study was to compare different surgical techniques in terms of morbidity and fertility results.

**Patients and methods:**

It is a retrospective study of interesting patients followed for infertility related to varicocele between January 2007 and December 2015. Three surgical techniques were compared: open inguinal surgery, antegrade sclerotherapy, and laparoscopy. Morbidity and pregnancy rate were assessed according to different techniques.

**Results:**

Post-operative complication rates were comparable (p = 0,94) between the 3 surgical techniques. An amelioration of sperm parameters has been noted in all operated patients, without statistical difference between the three techniques (p = 0,29 for the sperm concentration and p = 0,49 for the progressive mobility). Spontaneous pregnancy was better (p = 0,03) for patients who have had a varicocelectomy in a sub-inguinal way.

**Conclusion:**

All of the three surgical techniques used in this study showed an improvement of sperm parameters in an equal way with similar morbidity. However, the spontaneous pregnancy rate with open surgery was better.

## Highlights

• A varicocele is found in 15% of the male population and in 35% of men with infertility.
• Surgical treatment of a clinical varicocele improves spermogram parameters as well as the paternity rate.
• Open surgery should not be abandoned at the expense of sclerotherapy and laparoscopy.
• This technique was associated with a better paternity rate.

## 1 Introduction

A varicocele is determined as abnormal tortuous and dilated veins in the pampiniform venous plexus of the scrotal sac. This condition is found in 15% of the male population and in 35% of men with infertility [1,2]. This percentage increases to 81% in men with secondary infertility [3]. The cure of varicocele has been debated for several decades and multiple studies have shown that surgical treatment of a clinical varicocele improves spermogram parameters as well as the paternity rate [4]. The aim of our study was to determine the best surgical technique to operate on a varicocele in an infertile man.

## 2 Patients and methods

It was a retrospective, observational study conducted in a tertiary care center. Institutional Review Board approval was obtained (CEBM.EPS.HR/41/2020). Our data has been reported in line with the STROCSS criteria [5]. In this study, the authors confirmed that all methods were carried out under the relevant guidelines and regulations (Helsinki Declaration) under the number researchregistry 7857.

We retrospectively included all patients who followed up for infertility related to idiopathic varicocele between January 2007 and December 2015. Patients were divided into three groups according to the surgical technique performed: open surgery according to Ivanissevich, anterograde sclerotherapy according to Tauber, and laparoscopy. The choice of technique was randomized. Married patients followed for hypofertility whose wives had no cause for infertility and were under 40 years of age were included. All patients were explored by Doppler ultrasound. Preoperative spermogram abnormalities were confirmed by a second analysis performed at least 3 months after the first.

Operated patients were investigated by a spermogram performed six months after the surgical cure and the postoperative follow-up was at least one year. We did not include patients with another cause of infertility or whose operative indication was only the symptomatic nature of the varicocele. We excluded patients with azoospermia.

The statistical analyses were performed using SPSS version 20. Comparisons of two means on independent series were performed using Student's t-test. In case of invalidity of this test, the Mann-Whitney test and the Kruskal-Wallis test were used. The relationships between two quantitative variables were studied using the Pearson correlation coefficient. In case this test was not valid, we used the Spearman rank correlation coefficient. For all statistical tests, the significance level was set at 0.05.

## 3 Results

**Fig. 1:**
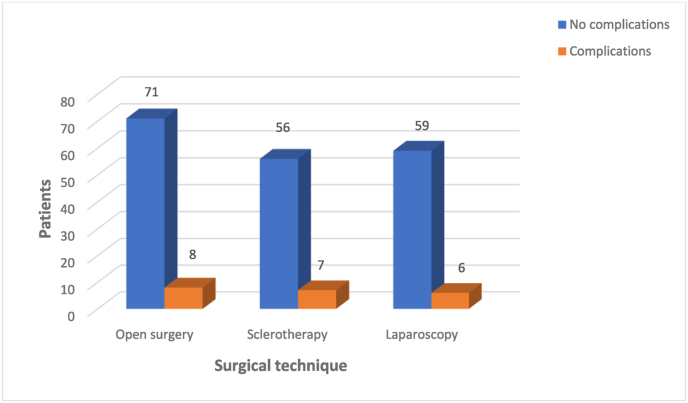
Postoperative complications for each surgical technique.

**Fig. 2:**
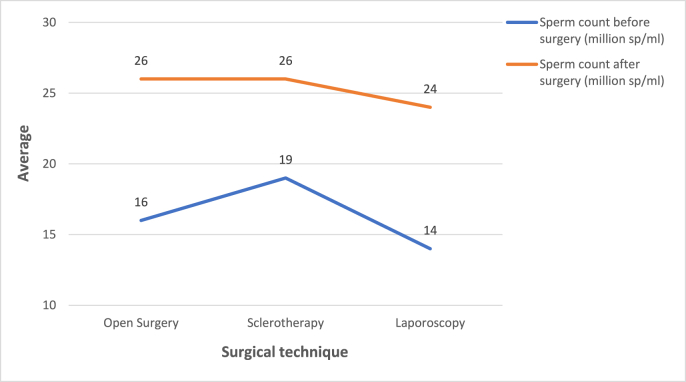
Evolution of sperm concentration after treatment according to surgical technique.

**Fig. 3:**
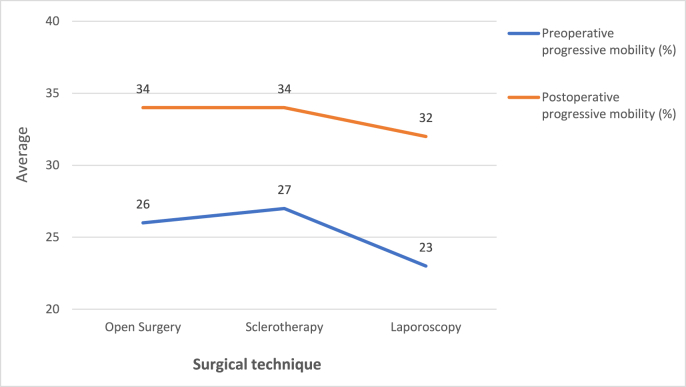
Evolution of progressive mobility after the treatment according to the surgical technique.

We included 207 patients. The average age was 35 years (22–54 years). Approximately 90% of the patients had no previous pathological history. The majority of the study population was asymptomatic (70%). We found oligoastheno-teratozoospermia in 53.14% of cases. About 71% of the patients had oligospermia and 88.28% had asthenospermia. Altered sperm morphology was found in 66.18% of cases. The classical open approach was chosen in 38.16% of patients (n = 79), antegrade sclerotherapy in 30.43% (n = 63) and laparoscopy in 31.4% (n = 65). Ten percent of the patients had complications such as recurrence of varicocele (4.8%), hydrocele (1.9%), orchitis (1.4%) or another complication (hematoma, wound infection). The postoperative complication rate was 10.12% for conventional surgery, 11.1% for sclerotherapy and 9.23% for laparoscopy. The lower rate observed with the latter technique was not statistically significant (p = 0.94) (Fig. 1). There was no significant difference in preoperative sperm concentration between the three groups of patients according to surgical technique (p = 0.54). This parameter improved in all patients after treatment but we found that none of the three techniques increased the postoperative sperm concentration more than the others (p = 0.29) (Fig. 2). Preoperative progressive sperm motility was equivalent in all three patient groups (p = 0.12). After treatment, this parameter improved in the entire population with no predominance of one surgical technique over the others (p = 0.49) (Fig. 3). The paternity rate was 48% for patients treated by inguinal ligation, 27% for those who had sclerotherapy and 26% for those who had laparoscopy. The best spontaneous pregnancy rate was significantly associated with open surgery (p = 0.03). The results are summarized in Table 1.

**Table 1:** Comparison of the three surgical techniques.

Variables	Open surgery	Sclerotherapy	Laparoscopy	P value
Preoperative sperm count (million/ml)	16	19	14	0.54
Postoperative sperm count (million/ml)	26	26	24	0.29
Preoperative progressive mobility (%)	26	27	23	0.12
Postoperative progressive mobility (%)	34	34	32	0.49
Paternity rate (%)	48	27	26	0.003
Complication rate (%)	10.12	11.1	9.23	0.94

## 4 Discussion

Several techniques have been described for the surgical treatment of varicocele [1,2]. Regarding the open approach, the retroperitoneal ligation according to Palomo (transverse skin incision two fingerbreadths medial and inferior to the homolateral anterosuperior iliac spine), the inguinal ligation according to Ivanissevich (transverse skin incision opposite the deep inguinal orifice), subinguinal ligation (transverse skin incision over the superficial inguinal orifice), microsurgery (using the same approaches and magnifying glasses or a surgical microscope) and antegrade sclerotherapy according to Tauber (skin incision at the root of the homolateral hemiscrotum) [3,4]. The laparoscopic approach allows the ligation of the spermatic veins a few centimeters from the internal inguinal orifice. Finally, radiological embolization (via the femoral or jugular vein) is mentioned [6,7].

Several studies have compared these different techniques. The ideal technique would have the fewest complications and the most improvement in the postoperative sperm count with a higher rate of spontaneous pregnancy. Postoperative complications of varicocele surgery can occur in 1%–5% of cases overall [8]. All surgical techniques may be associated with a risk of an infectious complication (orchitis, wall infection), hydrocele, recurrence of the varicocele or testicular atrophy. The single-centre retrospective study by Ghozzi et al. concluded that anterograde scrotal sclerotherapy was an equally effective, easy and reproducible technique in the treatment of idiopathic varicocele, with less morbidity and postoperative stay compared to laparoscopy and open surgery [9]. In a prospective randomized study, Simforoosh et al. compared open surgery with laparoscopy. After six months of follow-up, the rates of postoperative complications were similar [10].

Another randomized study of 298 infertile patients compared inguinal ligation (Ivanissevich), laparoscopy, and microsurgical sublingual ligation [11]. Postoperative complication rates were similar but microsurgery was found to have significantly less recurrence and hydrocele. Zucchi et al. compared the open inguinal approach with sclerotherapy in a randomized trial. The complication rates were comparable [12]. Sclerotherapy was also compared to laparoscopy in the randomized trial by Sautter. Early postoperative complications were significantly lower with sclerotherapy while recurrence rates were similar [13]. In a recent meta-analysis of different surgical techniques, microsurgery had the lowest rate of postoperative complications (0.44% hydrocele and 1.05% recurrence) while Palomo's technique had the highest (8.24% hydrocele and 14.97% recurrence) [14]. In our study, surgical treatment of varicocele improved sperm concentration and progressive motility. However, no surgical technique proved to be statistically significantly superior and the results were equivalent. Khouni's single-centre retrospective study found a statistically significant improvement in postoperative spermogram parameters in the group of patients treated with anterograde sclerotherapy (both sperm count and motility) compared with patients treated with open surgery and laparoscopy [15].

A prospective randomized trial by Simforoosh compared laparoscopic surgical treatment with open retroperitoneal surgery. After six months of follow-up, there was no difference in sperm parameters [10]. In the randomized Al-Said study (298 infertile patients with clinical varicocele), open inguinal surgery, laparoscopy, and sublingual microsurgery were compared. A significant improvement in sperm concentration, motility, and morphology was noted. However, the improvement in concentration and motility was significantly greater in patients treated with microsurgery [11]. Antegrade sclerotherapy was compared with microsurgery via the inguinal route in the treatment of clinical left varicocele in another randomized study. Postoperatively, sclerotherapy significantly increased the rate of progressive sperm motility [12]. In our work we found variability between the three different types of surgical techniques with regard to spontaneous pregnancy. This rate was 48% in men who had an open surgical cure. It was significantly higher (p = 0.03) than in patients who had antegrade sclerotherapy (27%) or laparoscopic cure (26%). A meta-analysis was performed to compare the surgical techniques for treating varicocele. The microsurgical approach was associated with the highest rate of spontaneous pregnancy (41.97%) while laparoscopy had the lowest rate (30.07%) [14]. In Al-Said's randomized study of open inguinal approach, laparoscopy and microsurgical sub-inguinal ligation, no significant difference was found in spontaneous pregnancy [11].

Several limitations of this study must be acknowledged before interpreting our findings. First, this study was based on a limited population at a single institution. Second, the retrospective descriptive design was not ideal for attaining study goals. Despite these limitations, our study showed the efficacy of three different surgical techniques in the management of varicocele in infertile men. Finally, a large-scale, multicenter, prospective study is needed to confirm these results.

## 5 Conclusion

Varicocele is significantly found in men with infertility. Although the mechanism of alteration of sperm parameters is not well understood, surgical treatment of clinical varicocele improves sperm concentration, motility, and morphology. In total, our study suggests that open surgery should not be abandoned at the expense of sclerotherapy and laparoscopy because, with a similar rate of postoperative complications, it was associated with a better paternity rate**.**

## Funding

We have any financial sources for our research.

## Provenance and peer review

Not commissioned, externally peer-reviewed.

## Sources of funding for your research

This research did not receive any specific grant from funding agencies in the public, commercial, or not-for-profit sectors.

## Ethical approval

Not applicable.

## Consent

Written informed consent was obtained from the patient for publication of this case report and accompanying images. A copy of the written consent is available for review by the Editor-in-Chief of this journal on request.

## Author contribution

Rahoui Moez, Yassine ouannes, Kais chaker and Mahdi Marrak: Data collection, Manuscript writing, Results discussion Kheireddine Mourad daly, Bibi Mokhtar, and Ahmed sellami: Manuscript writing and revision Ben rhouma sami and Nouira yassine: Paper revision.

## Registration of research studies

1. Name of the registry:: N/a
2. Unique identifying number or registration ID:: N/a
3. Hyperlink to your specific registration (must be publicly accessible and will be checked):: N/a

## Guarantor

Rahoui Moez is the guarantor of the study and accept full responsibility for the work and/or the conduct of the study, had access to the data and controlled the decision to publish.

## Declaration of competing interest

Authors do not report any conflict of interest.
